# P03.15. Developing a Health Qigong program for children: a 16-week curriculum

**DOI:** 10.1186/1472-6882-12-S1-P268

**Published:** 2012-06-12

**Authors:** C Wang, D Seo, R Geib, N Wroblewski, M Van Puymbroeck

**Affiliations:** 1 Indiana University Bloomington, Bloomington, USA; 2 Indiana University School of Medicine, Terre Haute, USA; 3 Monroe County YMCA, Bloomington, USA

## Purpose

With the increasing use of Traditional Chinese Medicine (TCM) in the West, Qigong has gained popularity for a variety of chronic health issues. However, there is a paucity of available literature that has systematically described the details and teaching strategies of Qigong. The purpose of this paper is three-fold: to demonstrate how to structure lesson content, to provide efficient teaching strategies, and to increase understanding of the underlying mechanisms of such programs’ potential benefits.

## Methods

A comprehensive literature review and a five-step process based on a theoretical framework (i.e., a formative evaluation approach) were used to develop a Health Qigong for Children program. The procedures include: (1) identifying the program, (2) developing educational strategies, (3) teaching pilot lessons, (4) consulting experts, and (5) drafting the curriculum.

## Results

Sixteen theme-based lesson plans were generated based on two traditional Health Qigong forms. Five promising teaching strategies were synthesized: (1) using theme-based lesson plans, (2) building mind-body connections, (3) balancing repetition and creativity, (4) interweaving pictures, stories, volunteers, and teamwork, and (5) involving parents and school teachers. Suggestions from an expert panel and student volunteers were solicited and incorporated into the program, that is, changing TCM-Based names for each Qigong movement into new names related to plants, animals, or interesting objects, and integrating some fun facts about the plants or animals into each lesson.

## Conclusion

The use of a theoretical framework was not only innovative but also effective. The Health Qigong for Children program has been successfully applied at several local elementary schools. Theme-based lessons and effective teaching strategies helped the Health Qigong program to be fun and age-appropriate for children. Suggestions from experts in a variety of fields strengthened the program design. The newly developed curriculum needs to be replicated with larger and various pediatric populations.

